# Expression of Concern: M6A‐ and m5C‐ Modified LncRNAs Orchestrate the Prognosis in Cutaneous Melanoma and m6A‐ Modified LINC00893 Regulates Cutaneous Melanoma Cell Metastasis

**DOI:** 10.1111/srt.70175

**Published:** 2025-05-05

H.‐Z. Shi, C.‐C. Tian, M.‐Y. Wu, L. Ma, J.‐F. Sun, and H. Chen, “m6A‐ and m5C‐ Modified LncRNAs Orchestrate the Prognosis in Cutaneous Melanoma and m6A‐ Modified LINC00893 Regulates Cutaneous Melanoma Cell Metastasis,” Skin Research and Technology 30, no. 7 (2024): e13842, https://doi.org/10.1111/srt.13842.

This Expression of Concern is for the above article, published online on 4 July 2024 in Wiley Online Library (wileyonlinelibrary.com), and has been issued by agreement between *Skin Research and Technology;* and John Wiley & Sons Ltd.

Although the stated aim of the article is to investigate the role of m6A‐ and m5C‐modified lncRNAs in the immune landscape and prognosis of melanoma, the presented results do not fully align with this objective. Specifically, the research objective outlined in the article could be interpreted as focusing on identifying lncRNAs that are direct subjects of epigenetic modifications; however, the study does not provide direct evidence demonstrating that the identified lncRNAs are direct targets of m6A‐ and m5C‐related genes.

During a post‐publication evaluation in collaboration with the authors, the editorial team concluded that, while the findings do not address the original research question, the methodology employed has led to the identification of lncRNAs whose expression levels vary in relation to the expression levels of m6A‐ and m5C‐related genes. As such, the presented results hold significance within a different biological framework and research perspective.

The authors also addressed discrepancies identified in Figure 2D, clarifying that labeling errors were responsible for the apparent inconsistencies between the results presented in the upper and lower panels, and provided documentation for the missing information related to the *ex vivo* and *in vitro* experiments in the updated Supporting Information material.

Nevertheless, as currently presented, several conclusions in the article may be overstated or potentially misleading. Therefore, the journal has decided to issue an Expression of Concern to inform and alert the readers.

